# De Novo Whole-Genome Assembly of the Swede Midge (*Contarinia nasturtii*), a Specialist of Brassicaceae, Using Linked-Read Sequencing

**DOI:** 10.1093/gbe/evab036

**Published:** 2021-02-28

**Authors:** Boyd A Mori, Cathy Coutu, Yolanda H Chen, Erin O Campbell, Julian R Dupuis, Martin A Erlandson, Dwayne D Hegedus

**Affiliations:** 1 Department of Agricultural, Food and Nutritional Science, University of Alberta, Edmonton, Alberta, Canada; 2 Saskatoon Research and Development Centre, Agriculture and Agri-Food Canada, Saskatoon, Saskatchewan, Canada; 3 Department of Plant and Soil Sciences, University of Vermont, Burlington, Vermont, USA; 4 Department of Entomology, University of Kentucky, Lexington, Kentucky, USA

**Keywords:** Cecidomyiidae, Diptera, insect pest, genomic resources, transcriptome, detoxification genes

## Abstract

The swede midge, *Contarinia nasturtii*, is a cecidomyiid fly that feeds specifically on plants within the Brassicaceae. Plants in this family employ a glucosinolate-myrosinase defense system, which can be highly toxic to nonspecialist feeders. Feeding by *C. nasturtii* larvae induces gall formation, which can cause substantial yield losses thus making it a significant agricultural pest. A lack of genomic resources, in particular a reference genome, has limited deciphering the mechanisms underlying glucosinolate tolerance in *C. nasturtii*, which is of particular importance for managing this species. Here, we present an annotated, scaffolded reference genome of *C. nasturtii* using linked-read sequencing from a single individual and explore systems involved in glucosinolate detoxification. The *C. nasturtii* genome is similar in size and annotation completeness to that of the Hessian fly, *Mayetiola destructor*, but has greater contiguity. Several genes encoding enzymes involved in glucosinolate detoxification in other insect pests, including myrosinases, sulfatases, and glutathione S-transferases, were found, suggesting that *C. nasturtii* has developed similar strategies for feeding on Brassicaceae. The *C. nasturtii* genome will, therefore, be integral to continued research on plant-insect interactions in this system and contribute to effective pest management strategies.

**Significance**

The swede midge is a serious pest of Brassicaceae, however, few genomic resources exist for the species. Here, we generated an annotated, scaffolded, genome assembly and used this genome to characterize adaptations needed for herbivore specialization on Brassicaceae. This assembly provides the foundation for applying genomics to the study of host plant manipulation and response to defenses and will undoubtedly have a significant impact on swede midge management.

## Introduction

Among insect herbivores, phytophagous flies within family Cecidomyiidae are considered to be among the most tightly linked to host plant biology because of their ability to manipulate growth of their hosts (Shorthouse et al. 2005). Cecidomyiid larvae secrete saliva onto plant tissues and feed via extra-oral digestion. Elements within the secretions cause swelling and deformation of plant tissue, and in some species, galls—structures composed of plant tissues created in response to stimuli produced by the gall-inducer (Mamaev 1975; Stone and Schönrogge 2003; Giron et al. 2016). Given that many cecidomyiids are agricultural and forestry pests (Hall et al. 2012), genome sequencing of these species can provide insight into host specialization, host manipulation, and traits that can inform pest management strategies.

The swede midge, *Contarinia nasturtii* (Kieffer) (Diptera: Cecidomyiidae), is native to Eurasia, and a significant pest of crops in the family Brassicaceae (Chen et al. 2011). Since arriving in North America (Hallett and Heal 2001), it has expanded its geographic range to include Eastern Canada, and Northeastern and Midwestern USA (Chen et al. 2011; Philips et al. 2017). In addition to feeding on cultivated crucifers, such as cabbage, cauliflower, broccoli (*Brassica oleraceae* L.), and canola (*B. napus* L., and *B. rapa* L.), it is found on a wide range of other Brassicaceae (Barnes 1946; Stokes 1953a, 1953b; Hallett 2007; Chen et al. 2009). Crop losses >80% have occurred due to *C. nasturtii* damage in broccoli (Hallett and Heal 2001) and canola (Hallett 2017), and larval feeding is particularly problematic on fresh vegetables as a single larva is capable of rendering them unmarketable (Stratton et al. 2018).

Investigation of the *C. nasturtii* genome can provide insight into host manipulation and adaptations needed for specialization on Brassicaceae that could have significant impacts on *C. nasturtii* management. Brassicaceae are well-known for their glucosinolate-myrosinase system, often called the “mustard-oil bomb” (Matile 1980; Barco and Clay 2019). Upon herbivore damage, glucosinolates are hydrolyzed by plant-derived myrosinases to form isothiocyanates, nitriles, and other compounds (Kissen et al. 2009), which are toxic to and/or deter many insect herbivores (Halkier and Gershenzon 2006). Nevertheless, some insect herbivores have developed strategies to avoid, sequester, or detoxify glucosinolates and their byproducts (reviewed in Winde and Wittstock 2011; Jeschke et al. 2016). Nitrile-specifier proteins in *Pieris* butterflies (Wittstock et al. 2004) and glucosinolate-specific sulfatases in the diamondback moth, *Plutella xylostella* (Ratzka et al. 2002) and cabbage stem flea beetle, *Psylloides chrysocephala* (Beran et al. 2018; Ahn et al. 2019) help prevent the formation of highly toxic glucosinolate-hydrolysis products. Other insects sequester glucosinolates and use them for defense in combination with insect-derived myrosinases (Jones et al. 2002; Kazana et al. 2007; Beran et al. 2014). Further, some insects employ general detoxification mechanisms, such as glutathione S-transferase (GST), to detoxify isothiocyanates and excrete them (Gloss et al. 2014; Beran et al. 2018), and additional mechanisms of detoxification are still being discovered (Friedrichs et al. 2020).

Overall, very little is known about the genomes of cecidomyiids. Until now, the Hessian fly, *Mayetiola destructor* (Say), a major pest of wheat (*Triticum aestivum* L.), was the only cecidomyiid genome sequenced (Zhao et al. 2015). Here, we describe an annotated genome assembly of *C. nasturtii* using linked-read sequencing, and identify several potential glucosinolate detoxification systems of this Brassicaceae specialist.

## Results and Discussion

### Genome Assembly, Annotation, and Quality Assessment

**Fig. 1.**
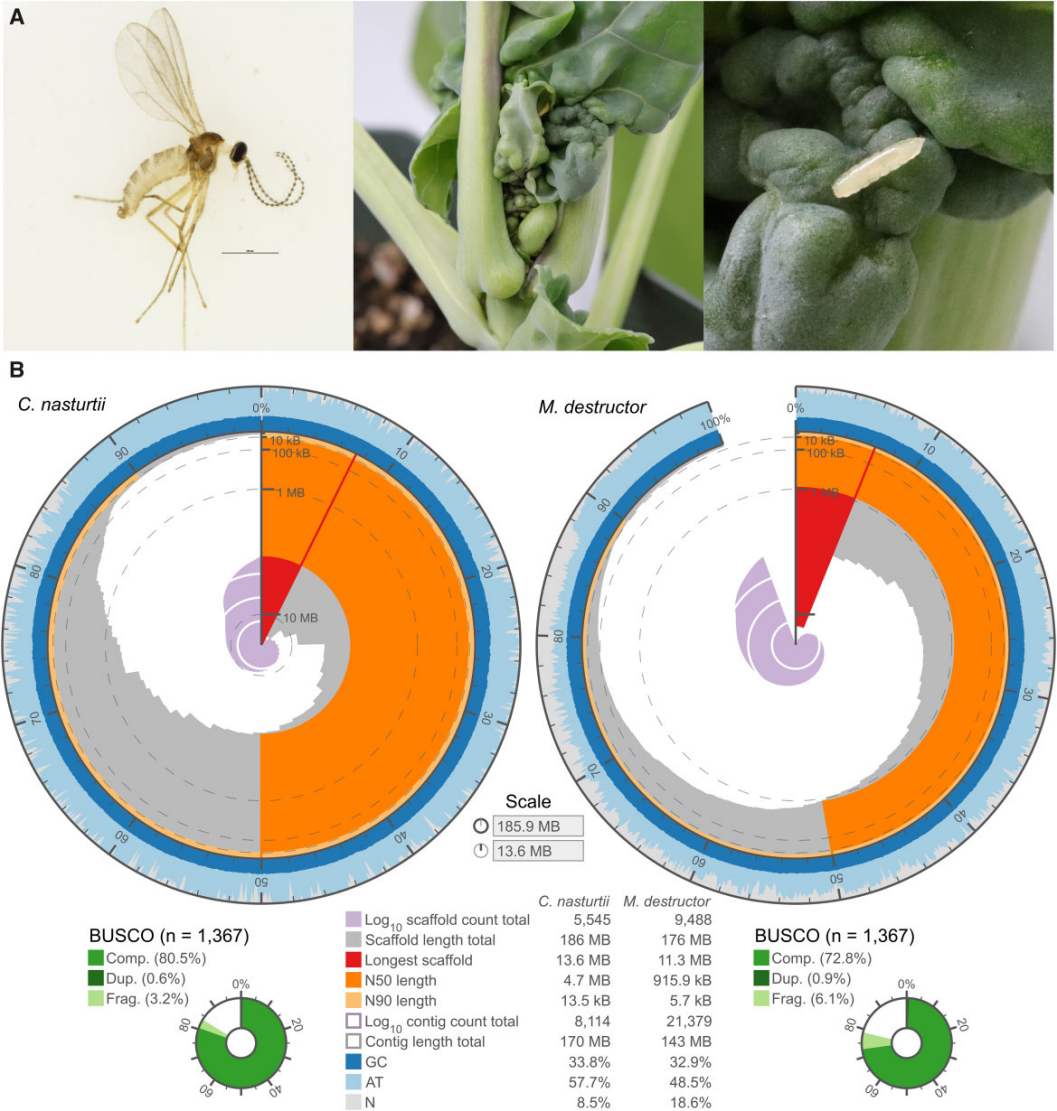
*Contarinia nasturtii* life stages (A) and comparison of continuity and completeness of the *C. nasturtii* (left) and *M. destructor* (right, Zhao et al. 2015) genome assemblies (B). (A): Adult male *C. nasturtii* (scale bar indicates 500 µm) (left); damage to canola, *Brassica napus* L. (va. AC Excel) caused by *C. nastrurtii* larval feeding (middle); and *C. nasturtii* larva (right). (B): Plots consist of scaffolds indicated by red and grey sections, sorted by descending length along the radius of each plot. The radius of each plot represents scaffold length with the scale marked at the vertical position (0%). The circumference of each plot (and percentage scale along the outside) indicates the percentage of the genome assembled into cumulative scaffolds, with N90, N50, and the longest scaffold indicated by light orange, dark orange, and red, respectively. Blue and light blue along the circumference represent relative GC/AT content. The cumulative number of scaffolds within a fraction of the genome is indicated by a purple spiral following the radial scale in thousands. Scaffolds of <1,000 bp were removed from the *M. destructor* assembly to match that of the *C. nasturtii* assembly. Complete (Comp.), duplicated (Dup.), and fragmented (Frag.) BUSCO annotations and assembly statistics are provided below.

In total, 88 million paired-end reads were generated from a linked-read library of a single *C. nasturtii* pupa. The highest quality de novo assembly (77 million reads, 57.2× raw coverage) had an effective coverage of 45× based on Supernova’s estimated genome size (supplementary table 1, Supplementary Material online). Based on *k*-mer distribution and analysis the sequencing error rate was 1.03% and heterozygosity was low (0.34%) (supplementary fig. 1, Supplementary Material online). The assembly consisted of 5,545 scaffolds with an N50 of 4.7 Mb (fig. 1), and was 185.9 Mb in total length, which was comparable to the average female genome size estimated by flow cytometry (female: 183.5 ± 0.9 Mb, male = 145.0 ± 0.5 Mb). The 174.9 Mb genome size estimated with *k*-mer spectra using Jellyfish/GenomeScope was complementary to these estimates (supplementary fig. 1, Supplementary Material online). Only 8.6% of the assembly was comprised of gaps. Completeness of the assembly using BUSCO revealed 79.9% of the core Insecta orthologs were complete and single copy, 0.6% complete and duplicated, 3.2% fragmented, and 16.3% missing (fig. 1, supplementary table 2, Supplementary Material online). Compared with *M. destructor* with a similar size genome, the *C. nasturtii* assembly was more contiguous (fig. 1, supplementary table 2, Supplementary Material online). These results suggest that linked-read sequencing is a viable option for minute insects and provides a cost-effective alternative to traditional approaches (Zhao et al. 2015). Additionally, linked-read technology allowed sequencing of a single individual with low heterozygosity without the need for inbreeding. This is of great practical importance for cecidomyiids as most are monogenous, that is, females generally lay eggs of only one sex (Barnes 1950; Dorchin and Freidberg 2004), which makes mating of offspring from a single individual impractical.

Annotation of the genome through NCBI’s eGAP identified 16,017 genes, with 14,889 containing protein-coding regions. This gene number is similar to other flies, including *Drosophila melanogaster* (Adams 2000), *Anopheles gambiae* (Holt et al. 2002), and *Musca domestica* (Scott et al. 2014). In total, 26,752 transcripts were annotated with a mean of 1.68 transcripts/gene; 23,265 (93.2%) transcripts were fully supported by experimental evidence. In addition to protein-coding genes, 1,789 noncoding RNAs were identified (1,537 fully supported), including tRNA, lncRNAs, and others. 84% of all aggregated reads from RNA-Seq libraries aligned to the genome indicating high reliability of the assembly. The full annotation report is available online (https://www.ncbi.nlm.nih.gov/genome/annotation_euk/Contarinia_nasturtii/100/, last accessed March 4, 2021). BUSCO analysis against the Insecta and Diptera gene sets suggested highly complete annotation with 98.8% and 92.0% complete (single and duplicated) matches, respectively (supplementary table 2, Supplementary Material online). Compared with the original gene set of *M. destructor*, the *C. nasturtii* RefSeq gene set is on average 7.3% more complete (across Insecta and Diptera odb10) (supplementary table 2, Supplementary Material online). Comparison of *k*-mer spectra using KAT indicated that haploid representation of the assembly was successful and free of diploid content (supplementary fig. 2, Supplementary Material online). Prior to annotation, WindowMasker masked 30.01% of the assembly (supplementary table 3, Supplementary Material online); this level of repetitive sequences is less than that observed (52%) in *M. domestica* and several other insect species (Scott et al. 2014). *k-*mer analysis estimated 13.9% of the genome is made up of repetitive content (supplementary fig. 1, Supplementary Material online), which is similar to the repeat content in the *M. destructor* genome (12%) (Zhao et al. 2015). Despite differences in estimates between the two programs likely due to assembly vs. raw read inputs, the respective comparison to other species (e.g. Scott et al. 2014, Zhao et al. 2015) supports the high quality of this assembly.

### Identification of Glucosinolate Detoxification Systems

**Fig. 2.**
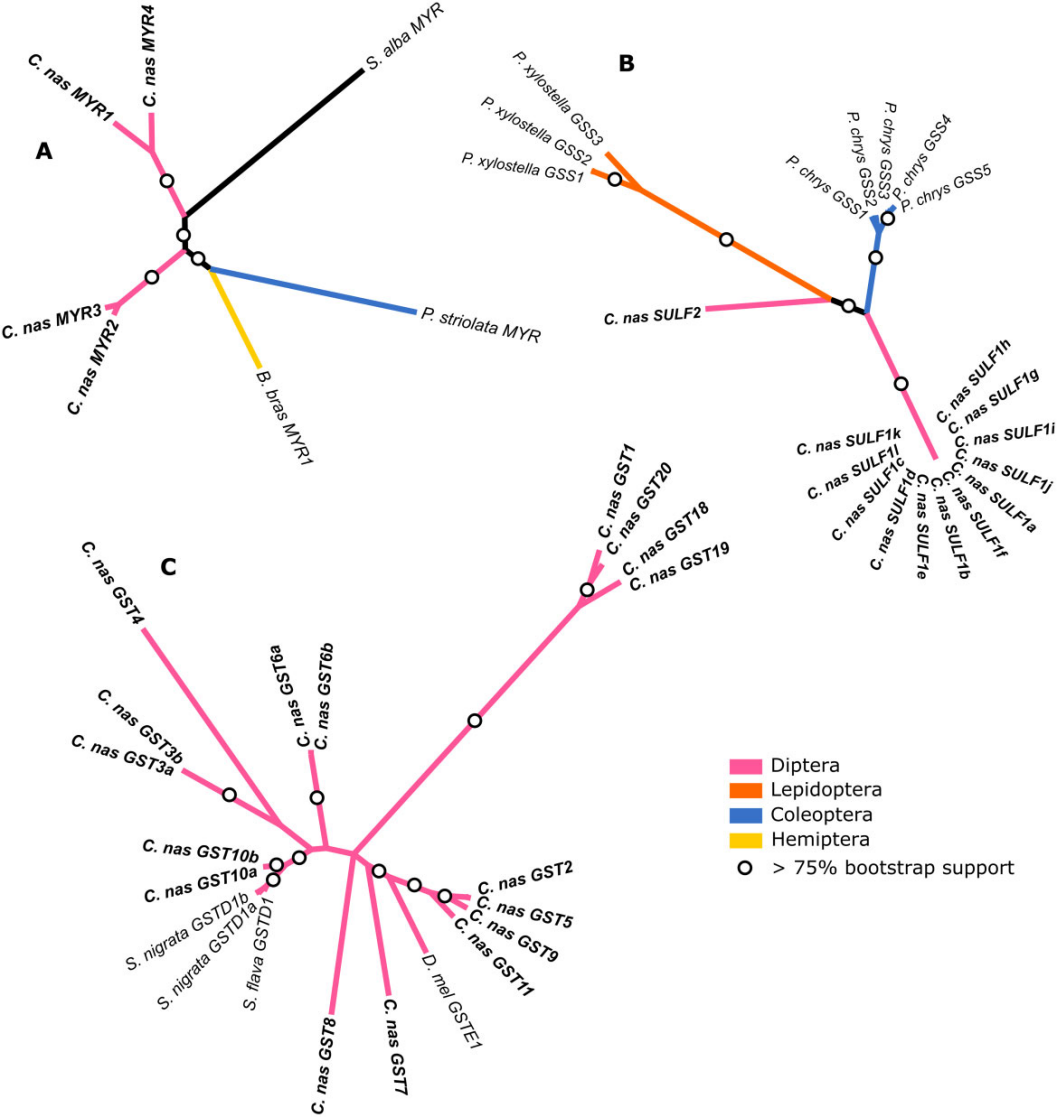
Unrooted maximum likelihood consensus trees of amino acid sequences for enzymes involved in glucosinolate detoxification that were identified in the *C. nasturtii* RefSeq annotations, with additional sequences from Genbank. Myrosinases are shown in panel (A), sulfatases in (B), and GSTs in (C). The color of each branch indicates the insect Order of the sequences in each phylogeny; the only noninsect taxon, *S. alba* (Brassicaceae) is pictured in black in (A). Branch labels indicate the taxon name followed by the gene ID, and the following taxon names are abbreviated on the trees: *C. nas*, *C. nasturtii*; *B. bras*, *B. brassicae*; *D. mel*, *D. melanogaster*; and *P. chrys*, *P. chrysocephala*. *Contarinia nasturtii* sequences are indicated in bold text.

Several genes encoding components of glucosinolate detoxification systems functionally characterized in other insects were identified in the *C. nasturtii* genome. Both *Phyllotreta striolata* (Beran et al. 2014) and *Brevicoryne brassicae* (Kazana et al. 2007) are capable of sequestering glucosinolates and possess myrosinases that are distinct from plant myrosinases (Jones et al. 2002, Beran et al. 2014). The four full-length *C. nasturtii* myrosinase genes identified were also unique from plant myrosinases (fig. 2, supplementary fig. 3 and supplementary table 4, Supplementary Material online). *C. nasturtii* myrosinase-2 and 3 clustered with those from *P. striolata* and *B. brassicae*, but not with *C. nasturtii* myrosinase-1 and 4 (fig. 2*A*). The *C. nasturtii* myrosinases contain conserved glucose-binding and catalytic sites, as in *P. striolata* and *B. brassicae* myrosinases, and are likely functional (supplementary fig. 3, Supplementary Material online).

Sulfatases have evolved in insects that feed on Brassicaceae plants to modify glucosinolates so they are no longer recognized by myrosinases and converted into toxic derivatives (Jeschke et al. 2016). Insect sulfatase was first discovered in *P. xylostella* (Ratzka et al. 2002) and, more recently, in *P. chrysocephala* (Beran et al. 2018; Ahn et al. 2019). No *C. nasturtii* genes were annotated as encoding sulfatases. However, Ahn et al. (2019) found five arylsulfatase-like enzymes with sulfatase activity in *P. chrysocephala* and two genes encoding arylsulfatase-like enzymes were found in *C. nasturtii*, one of which had five isoforms (fig. 2*B*, supplementary table 5, Supplementary Material online). The two putative *C. nasturtii* arylsulfatase-like enzymes clustered with sulfatases from *P. chrysocephala*. Furthermore, there was high conservation in amino acid residues of *C. nasturtii* arylsulfatase-like enzymes and both possessed sulfatase signature features and catalytic residues (supplementary fig. 4, Supplementary Material online). Interestingly, sulfatases in *P. xylostella* and *P. chrysocephala* contain a signal peptide and are secreted into the midgut (Ratzka et al. 2002; Ahn et al. 2019), while those in *C. nasturtii* lack a signal peptide indicating they are not secreted (supplementary table 5, Supplementary Material online). As the feeding behavior of *P. xylostella* and *P. chrysocephala* are markedly different from larval *C. nasturtii*, it is possible that glucosinolate detoxification occurs after uptake by the cell or that *C. nasturtii* sulfatases have a different biological function.

GSTs are known for their role in xenobiotic detoxification and are represented by several classes within the larger family (Ranson et al. 2001), some of which play a role in glucosinolate detoxification (Gloss et al. 2014, 2019). Gloss et al. (2014) found the Brassicaceae-specialist-drosophilids *Scaptomyza flava* and *S. nigrita* use delta-class glutathione S-transferase 1 (GSTD1) for glucosinolate detoxification. Twenty genes encoding GST-like genes were found in the *C. nasturtii* genome, 17 of which were complete and were within the expected size range (fig. 2*C*, supplementary table 6, Supplementary Material online). *C. nasturtii* GST10a and GST10b clustered with GSTD1s from *S. flava* and *S. nigrita* (fig. 2) and residues in the aromatic zipper motif (H-site and α8-helix) were well-conserved (supplementary fig. 5, Supplementary Material online); these were identified by Gloss et al. (2014) as important for isothiocyanate detoxification. Recently, Gloss et al. (2019) found epsilon class GSTs (GSTE) were also involved in glucosinolate detoxification and four *C. nasturtii* GSTs clustered with *D. melanogaster* GSTE1 (fig. 2). Based on our combined results, *C. nasturtii* myrosinases, arylsulfatases, and GSTs should be explored further to examine their roles in glucosinolate detoxification.

### Conclusions

The sequencing of the *C. nasturtii* genome provides the foundation necessary to explore plant-gall insect interactions at the molecular level. Due to the intimate interaction between cecidomyiid larvae and their host plants during feeding, they are thought to follow a gene-for-gene model of coevolving “effectors” (Hatchett and Gallun 1970), which was further supported in *M. destructor* based on analysis of its genome (Zhao et al. 2015). These gene-for-gene interactions have yet to be explored in *C. nasturtii*; however, we have begun to investigate insect adaptation in this system by identifying potential glucosinolate detoxification systems characterized in other insects. Given the nature of *C. nasturtii* infesting plants in the Brassicaceae, which have formidable defenses in the form of glucosinolate-myrosinase systems, this genome should foster exploration of both broader questions of insect-plant coevolutionary dynamics and potential use of these genes in pest management.

## Materials and Methods

### Genome Sequencing, Assembly, and Size Estimation

DNA was extracted from a single *C. nasturtii* pupa according to the 10× Genomics’ (Pleasanton, CA) “DNA extraction from single insects” protocol with homogenization by razor blades (details in Supplementary Material online). DNA concentration was adjusted to 0.65 ng/µl and loaded onto a Chromium Genome Chip. Libraries were prepared using the Chromium Genome Library & Gel Bead Kit v.2 (10× Genomics) and Chromium controller according to manufacturer’s recommendations, but with additional shearing (Major et al. 2020). The library was quantified by qPCR using a Kapa Library Quantification Kit (Kapa Biosystems-Roche) and sequenced on a partial lane of NovaSeq6000 (Illumina, San Diego, CA) with paired-end 150 bp reads.

Raw reads were demultiplexed with *mkfastq* in Supernova v2.1.1 (10× Genomics) by the UC Davis Genome Centre (details in Supplementary Material online). Genome characteristics from the *k*-mer distribution of debarcoded raw reads were estimated with Jellyfish v2.2.3 (Marçais and Kingsford 2011) and GenomeScope v1.0 (Vurture et al. 2017) (*k *=* *21, *k*-mer coverage cutoff = 10,000).

A de novo genome assembly was constructed with *mkoutput* (style = pseudohap2) in Supernova v2.1.1. Reads were subsampled to create and compare several assemblies (48–60× coverage) (supplementary table 1, Supplementary Material online). The highest quality assembly (based on a balance between scaffold N50, phase block N50, contig N50, and predicted genome size) was obtained with 77 million randomly selected reads (∼57×) (56× recommended) and was submitted to NCBI (GCA_009176525). During processing and quality checks, NCBI identified 1,115 sequences that may have originated from bacterial contaminants, ecto-, or endo-symbionts; these were masked in the updated assembly AAFC_1.1 (GCA_009176525.2) (supplementary table 7, Supplementary Material online).

Genome size was estimated by flow cytometry from DNA isolated from individual adult male (*n* = 13) and female (*n* = 10) *C. nasturtii* heads. The head of a female *Drosophila virilis* was used as an internal standard (1 C = 328 Mb genome) (Johnston et al. 2019).

### Genome Annotation and Quality Assessment

Structural and functional annotation of genes was conducted with NCBI’s Eukaryotic Genome Annotation Pipeline (eGAP) v.8.3 (Thibaud-Nissen et al. 2013). To aid in annotation, RNA-Seq was conducted on pooled samples of each *C. nasturtii* life stage (eggs, first-third instar larvae, pupae, and adult males and females) (NCBI SRA: SRX6853817-SRX6853823) (details in Supplementary Material online). Additional transcripts available from salivary glands (NCBI SRA: SRS5439046) were also used. Prior to annotation, eGAP uses RepeatMasker (http://www.repeatmasker.org, last accessed March 4, 2021) and/or WindowMasker (Morgulis et al. 2006) to mask repeats in the genome assembly; after which, eGAP uses the RNA-Seq data and several NCBI RefSeq protein sets to inform gene model prediction. The full eGAP process can be accessed online at: https://www.ncbi.nlm.nih.gov/genome/annotation_euk/process/#process (last accessed March 4, 2021).

BUSCO (Benchmarking Universal Single-Copy Orthologs) v.3.0.2 was used to assess the completeness of the genome/annotated gene set against the Insecta and Diptera odb10 data sets (Simão et al. 2015; Waterhouse et al. 2018). BUSCO results were compared with the *M. destructor* genome (GCA_000149195.1) and the original gene set (OGS1.0) from the i5K initiative [https://i5k.nal.usda.gov/data/Arthropoda/maydes-(Mayetiola_destructor)/, last accessed March 4, 2021]. BlobToolKit was used to visualize quality metrics of the *C. nasturtii* and *M. destructor* genomes (Challis et al. 2020). To validate the assembly and ensure it was largely free of diploid content, KAT v2.4.1 (Mapleson *et al.* 2016) in Comp mode (with default settings, *k *=* *27) was used to compare *k*-mer spectra of raw reads (debarcoded) to those of the assembly.

### Identification of Glucosinolate Detoxification Systems

Genes encoding elements of glucosinolate detoxification systems that had been functionally characterized in other insects (i.e., myrosinases, sulfatases including arylsulfatase, and GSTs) were identified from the *C. nasturtii* RefSeq gene set annotations and the genome assembly and manually curated (details in Supplementary Material online, supplementary tables 4–6, Supplementary Material online). SignalP 5.0 (Armenteros et al. 2019) and TMHMM 2.0 (Krogh et al. 2001) were used to predict signal peptides and transmembrane domains, respectively. ProtoParam (Gasteiger et al. 2005) was used to predict molecular weight and isoelectric points for each protein. Amino acid sequences were aligned in MAFFT (https://mafft.cbrc.jp/alignment/server/, last accessed March 4, 2021; L-INS-i algorithm, Mafft homologs—on). Maximum likelihood phylogenies for genes of interest were constructed using IQ-TREE’s web server (Trifinopoulos *et al.* 2016) and resulting extended consensus trees were visualized with FigTree 1.4.4 (Rambaut and Drummond 2010) (details in Supplementary Material online).

## Supplementary Material

evab036_Supplementary_Data
