# Supplementary material for: De Novo Whole-Genome Assembly of the Swede Midge (Contarinia nasturtii), a Specialist of Brassicaceae, Using Linked-Read Sequencing
Source: Genome Biol Evol. 2021 Feb 28;13(3):evab036. doi: 10.1093/gbe/evab036 (PMC8011032; doi:10.1093/gbe/evab036)
Supplement: evab036_Supplementary_Data [file evab036_supplementary_data.zip › Mori et al. - Supplemental Materials and Figures.docx]

**Title**

*De novo* whole genome assembly of the swede midge (*Contarinia nasturtii*), a specialist of Brassicaceae, using linked-read sequencing.

**Authors**

Boyd A Mori, Cathy Coutu, Yolanda H Chen, Erin O Campbell, Julian R Dupuis, Martin A Erlandson, Dwayne D Hegedus

**Supplementary Materials**

*Insects*

*Contarinia nasturtii* were obtained from a colony maintained at Agriculture and Agri-Food Canada (Saskatoon, SK, Canada). Midges were reared as previously described (Liu et al. 2019). Ten *C. nasturtii* pupae were flash-frozen in liquid nitrogen 24 hours after pupation and submitted to the UC Davis Genome Center (CA, USA) for extraction and sequencing.

*De novo genome assembly*

Raw reads were demultiplexed with *mkfastq* in Supernova v2.1.1 (10x Genomics) by the UC Davis Genomics Core (Davis, CA). Upon receipt, we examined read quality and checked for adaptor contamination with FastQC v0.11.5 (Andrews 2010). No issues were noted and no further trimming was conducted. To assemble a *de novo* genome using Supernova v2.1.1, the recommended practice is to subsample the number of reads to obtain ~56x raw genome coverage (10x Genomics). To determine the optimal coverage for *C. nasturtii*, assemblies with 48-70X coverage were created and their quality assessed using summary statistics (Scaffold N50, phase block N50, contig N50, and predicted genome size as output by Supernova). Supernova default settings were used, with style set to pseudohap2 and a minimum scaffold size set to 1000 bp. Completeness of each genome assembly was also assessed with BUSCO v1.22 against the Arthropoda odb10 dataset (Simão et al. 2015; Waterhouse et al. 2018). The highest quality genome assembly was defined as the one that balanced the longest N50 values (with emphasis on the contig N50) with the most core Arthropoda genes (Supplementary Table 1).

*RNA-Sequencing*

Pooled samples of each *C. nasturtii* life stage [(eggs, n = 2056); first, second and third instar larvae (n = 100 each); pupae (n = 100); adult males and females (n = 100 each)] were collected. Total RNA was extracted using the TRIzol reagent and protocol (Invitrogen) and purified using an Illustra^TM^ RNAspin Mini Kit with DNase treatment (GE Healthcare Life Sciences). Paired-end, 125-bp, sequencing libraries were generated using the Illumina TruSeq Stranded RNA Kit (Illumina), and sequenced on a lane of Illumina HiSeq 2500 at the National Research Council of Canada (Saskatoon, SK, Canada). Reads were trimmed for quality and adaptor sequences using Trimmomatic v.0.30 (Bolger et al. 2014) (parameters: LEADING: 5 TRAILING:5 SLIDINGWINDOW: 4:15 MINLEN:36) before deposition in the NCBI Short Read Archive (SRX6853817-SRX6853823).

*Removal of bacterial contaminants*

NCBI identified potential bacterial contamination during the eukaryotic genome annotation process. Using a mixture of k-mer, nucleotide BLAST, and a variation of a protein BLAST approaches, 6.9 Mbp of contaminating sequence were identified. Only a single contig was >100 kb (VYII01001287.1, at 3.6 Mbp) with the majority being shorter than 10 kb, which is typical of such contaminants. In total, 1,115 contigs were suppressed in the original assembly (Supplementary Table 2) and the assembly was updated (AAFC_1.1, GCA_009176525.2).

*Comparison of* Mayetiola destructor *and* Contarinia nasturtii *genome assemblies*

The *Contarinia nasturtii* BUSCO results were compared to the *M. destructor* genome (**GCA_000149195.1) and the original gene set (OGS1.0) from the i5K initiative (**<https://i5k.nal.usda.gov/data/Arthropoda/maydes-(Mayetiola_destructor)/>**). However, to compare quality metrics between the two genomes** with the Blobtoolkit (**Challis et al. 2020),** all contigs <1,000 bp from the *M. destructor* assembly (GCA_000149195.1) **were first removed** as the *C. nasturtii* genome assembly specified a minimum contig length of 1,000 bp. Allowing contigs <1,000 bp in the *M. destructor* assembly would have skewed the quality metrics.

*Identification of glucosinolate detoxification genes, manual curation*, *and phylogenetic analyses*

For identification and characterization of genes involved in glucosinolate detoxification, protein sequences of myrosinases from *Phyllotreta striolata*, *Brevicoryne brassicae*, and the mustard plant, *Sinapis alba*; glucosinolate sulfatases from *Plutella xylostella*; arylsulfatases from *P. chryosocephala*; and, delta class GSTs (GSTDs) from *Scaptomyza flava* and *S. nigrita* were compiled (Supplementary Table 3-5). Recently, epsilon class GSTs (GSTEs) were found to be involved in glucosinolate detoxification in *S. flava* (Gloss et al. 2019). The sequences for the *S. flava* GSTEs are not in the public domain at this time; however, the GSTEs in *S. flava* were homologous to GSTEs in *Drosophila melanogaster* (Gloss et al. 2019), therefore, a representative GSTE protein sequence from *D. melanogaster* was also included (Supplemental Table 5). Next, the RefSeq *C. nasturtii* annotated gene set was searched via keywords including ‘myrosinase’, ‘glucosinolate sulfatase’, ‘arylsulfatase’, and ‘glutathione S-transferase’ and the protein sequences retained. Finally, a TBLASTN (e-value: 1e^-15^) search was used to query the above complied protein sequences against the *C. nasturtii* genome assembly (GCF_009176525.2) in CLC Genomics Workbench v20.0.4 (Qiagen). Each hit region was manually inspected. If a gene was already annotated, based on the automated eukaryotic genome annotation pipeline (eGAP) (NCBI), its protein sequence was compared to the other *C. nasturtii* genes in that gene family. Occasionally, the TBLASTN search resulted in a putative gene region which was not annotated by eGAP, for both these gene regions and those annotated by the eGAP pipeline, expression levels were confirmed and the exon locations determined by mapping one set of RNAseq reads from each *C. nasturtii* life stage to the genome in CLC Genomics Workbench parameters: Masking mode = No masking; Match score = 1; Mismatch cost = 2; Cost of insertion and deletions = linear gap cost; Insertion cost = 3; Deletion cost = 3; Similarity fraction = 0.9). A final TBLASTX of all putative *C. nasturtii* detoxification proteins against the genome was performed to detect any remaining genes missed in the first BLAST.

For each protein family, a multiple sequence alignment (CLUSTAL) was generated to inspect genes for a late start, early stop, or insertion or deletion (relative to other family members) to confirm start, stop, and splice sites in CLC Genomics Workbench. Truncated genes were inspected, to determine if gaps or scaffold ends were present in the genome sequence. If a protein differed from other family members, annotated splice sites were confirmed using mapped RNAseq reads. Finally, BLASTN was used to check if short scaffolds were nearly identical to other regions in the genome, to determine if they are possible allelic variants. Through this process only one additional complete gene was identified and corrected (a myrosinase which was originally annotated as a pseudogene), and two partial genes which were annotated as other genes were identified as putative myrosinases (Supplemental Table 3). Annotation notes are included in Supplementary Tables 3-5, and all *C. nasturtii* protein sequences are provided in Supplementary Table 8.

Only complete genes, within the expected size range, were used for phylogenetic analyses. Amino acid sequences were aligned in MAFFT (<https://mafft.cbrc.jp/alignment/server/>; L-INS-i algorithm, Mafft homologs – on). Maximum likelihood phylogenies for genes of interest were constructed using the IQ-TREE web server (Trifinopoulos *et al.* 2016) with 1000 replicates of ultrafast bootstrapping (Hoang *et al.* 2018) and SH-aLRT branch testing (Guindon *et al.* 2010); model selection was conducted within IQ-TREE using ModelFinder (Kalyaanamoorthy *et al.* 2017). The resulting extended consensus trees were visualized with FigTree 1.4.4 (Rambaut and Drummond 2010).

**Supplementary Figures**

**
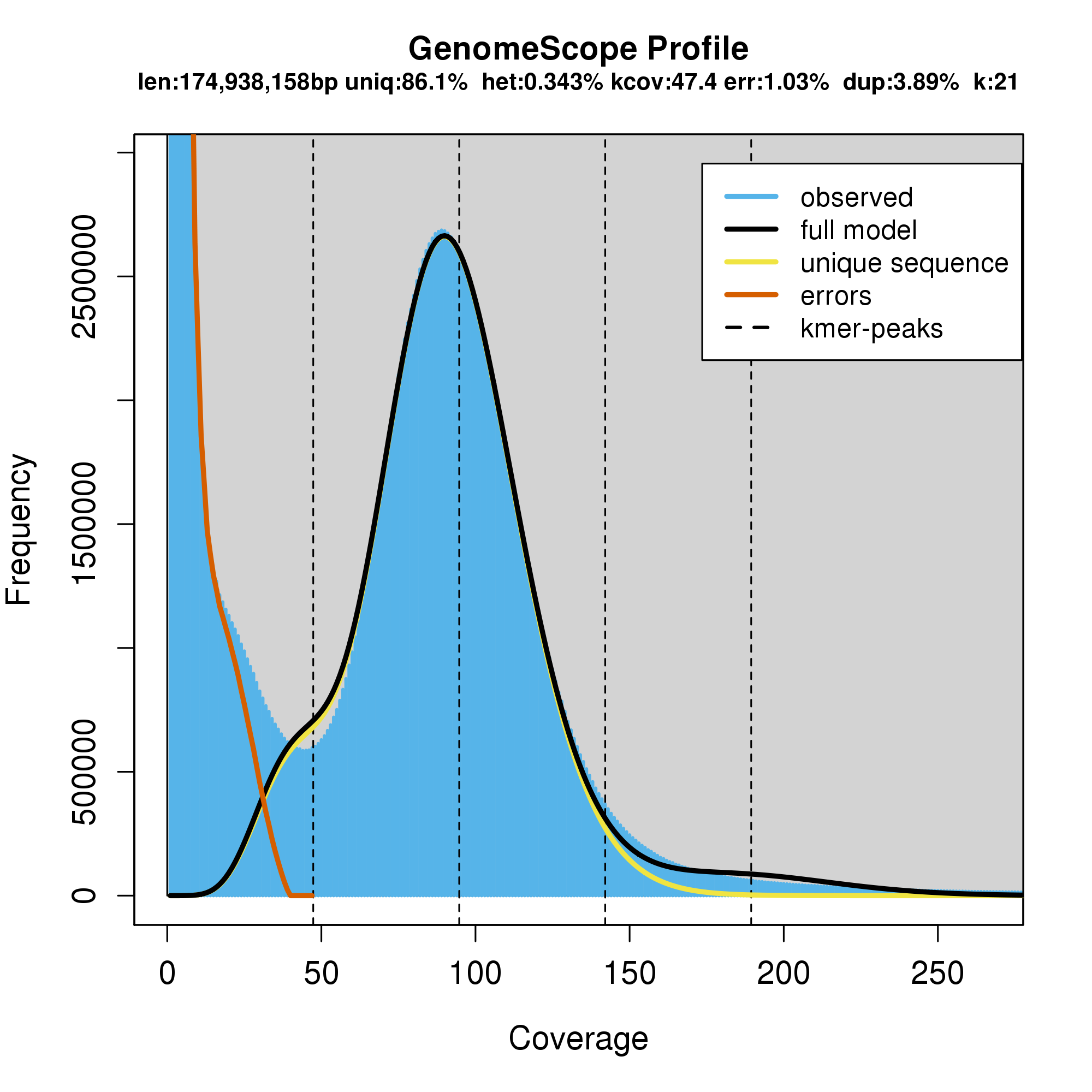
**

**Supplementary Figure 1:** *k*-mer profile (*k* = 21) of the *C. nasturtii* genome debarcoded raw reads as calculated by Jellyfish and GenomeScope. Blue indicates observed distribution, and black, yellow, and red lines indicate modeled *k*-mer distributions of the full genome, unique sequences, and sequencing errors, respectively. Statistics for assembly length, unique sequences, heterozygosity, *k­*-mer coverage, error, and duplicated sequences provided at the top.


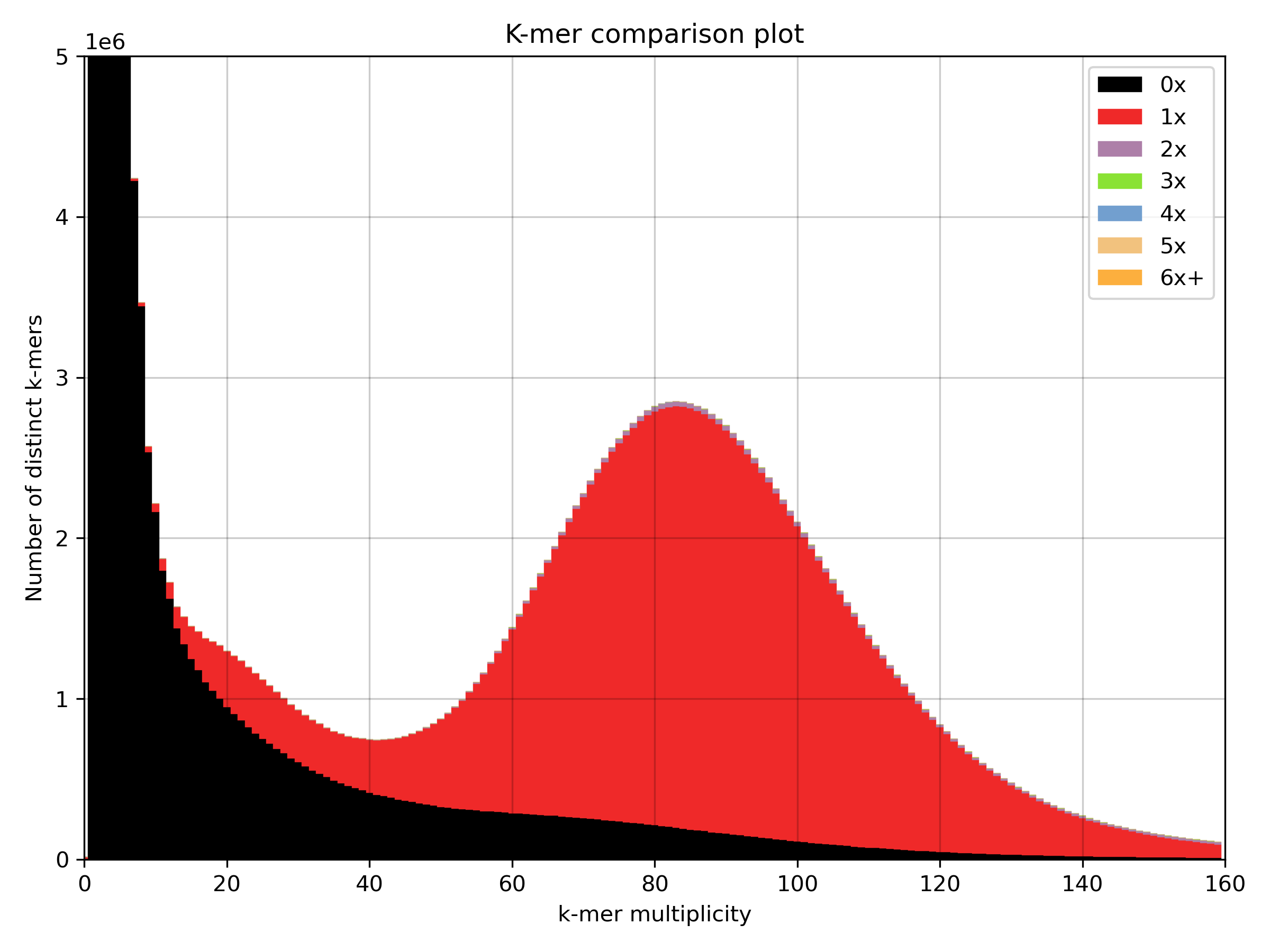


**Supplementary Figure 2:** *k*-mer spectra of raw reads compared to the genome assembly with color representing copy number (black indicates content not present in the assembly).

◊

PstrMyr 1 ***MQ--QTIAFVLVLL-------------QFAFNADG***ALKNNGQFPRNFLFGAATASYQIEG
BbraMyr 1 --------------------------------------MDYKFPKDFMFGTSTASYQIEG
CnasMyr2 1 ***M---NFYQNLGIFL-------VIFFYL----QNGW***CEKVNRKFPSDFLFGVGSSAYQVEG
CnasMyr3 1 ***M---KFYQSLAIAS-------VTVLYL----QNGW***CEKLNPMFPPDFLFGVGSSAYQVEG
CnasMyr1 1 ***MFLSRLIYIFTIFI---------VE------IVVA***KNGSSQHFPENFLFGVSSSAYQIEG
CnasMyr4 1 ***MRSSKLICIIFIFI---------SSGS----A***SPPKSESNRYFPPDFLFGAATSAYQVEG
SalbMyr 1 --------------DEEITCQENNPFT----CGNTDGLNSSSFEADFIFGVASSAYQIEG


PstrMyr 46 AWNEDGKGQSTWDEFTHRIPSPV-TNNDTGDIACDHYHRYKEDIKMAADLGLQAYRFSIS
BbraMyr 23 GWNEDGKGENIWDRLVHTSPEVI-KDGTNGDIACDSYHKYKEDVAIIKDLNLKFYRFSIS
CnasMyr2 47 GWNKHGKGESIWDVLTHQYPEKM-TDKSNADVTSDSYHNWRRDVEMVRELGVDIYRFSIS
CnasMyr3 47 GWNKHGKGESIWDVLTHQYPEKM-KDKSNADVTSDSYHNWRRDVEMVRELGVDIYRFSIS
CnasMyr1 46 GWDSDGKGPSIWDEFTHLHPEKI-VDRQNGDVGANSYEYYLDDVEAVKSLNMNFYRFSIS
CnasMyr4 48 GWDSDGKGPSIWDDFVHSYSENI-LDHQNADVGANSYEYYLDDIEALKSLNMNFYRFSIS
SalbMyr 43 TI---GRGLNIWDGFTHRYPDKSGPDHGNGDTTCDSFSYWQKDIDVLDELNATGYRFSIA

 ◊
PstrMyr 105 WPRVLPNGY-ADTINEKGMQFYKDLVDEIVKYGMVPVCTLFHWDLPLKLYE-NGIDWRNE
BbraMyr 82 WARIAPSGV-MNSLEPKGIAYYNNLINELIKNDIIPLVTMYHWDLPQYLQD-LG-GWVNP
CnasMyr2 106 WTRILPNGF-VTNVNKHGIRYYNNLINELLRYNITPMVTIYHWDLPARLQE-LG-GWTNP
CnasMyr3 106 WTRLLPNGF-VTNVNKHGIAYYNNLINELLRHNITPMVTIYHWDLPARLQE-LG-GWTNP
CnasMyr1 105 WTRILPTGD-LTQVNEKGIEYYNKIINKLIEYKIEPMVTMYHFDLPAHLQL-LG-GFANS
CnasMyr4 107 WSRILPTGD-ITQINEKGIEYYNKLINALIENEIQPMVTMFHFDLPQKLQL-FG-GFTNS
SalbMyr 100 WSRIIPRGKRSRGVNQKGIDYYHGLIDGLIKKGITPFVTLFHWDLPQTLQDEYE-GFLDP

 ◊▼
PstrMyr 163 KIIDIFVAYSRLMIQNL-PKVGYWSTINEPRVHCLRSYGDGKHAPGIAE-----------
BbraMyr 139 IMSDYFKEYARVLFTYFGDRVKWWITFNEPIAVC-KGYSIKAYAPNLNL-----------
CnasMyr2 163 AIIDYVTDYAKILFEQFGDRVKIWTTINEPWHICEQAYGVDFMAPAMNF-----------
CnasMyr3 163 VIIDYVTDYAKILFEQFGDRVKIWTTINEPWHICEQGYGVDIMAPAMNF-----------
CnasMyr1 162 AIVQYFEEYANLLFNRFGDRVKYWITINEAAEFCVKGYGGKIHAPGINA-----------
CnasMyr4 164 VIVRYFEEYANLLYNRFGDRVKYWITINEPNIFCKYGYGTNKNAPGMNA-----------
SalbMyr 159 QIIDDFKDYADLCFEEFGDSVKYWLTINQLYSVPTRGYGSALDAPGRCSPTVDPSCYAGN
 ● ●

PstrMyr 211 SGIADYQCSYVILKAHAKAYRMYKKEF-PHYKAPFGIVIDCQWYVPAT-NSS-EDIAAAE
BbraMyr 187 KTTGHYLAGHTQLIAHGKAYRLYEEMFKPTQNGKISISISGVFFMPKNAESD-DDIETAE
CnasMyr2 212 PGIPSYLCGHNLLKAHAEIVHLYRERFQPTQKGKIGITTDTSYTQAKT-DSN-DDKIASE
CnasMyr3 212 PGIPSYLCGHNVLKAHAEIVHLYRERFQPTQKGKIGITIDTSYAQAKT-DSD-DDKVASE
CnasMyr1 211 HGKGEYLCVHNALKSHAVAYHLYKREYYNQYKGQVGIALDSSFFYSDS-----NDTVVVN
CnasMyr4 213 SGVADYLCIHNALKAHAVAYHLYKDKFYNQFKGRIGICLLSYFFYSDS-----NDAIAVD
SalbMyr 219 SSTEPYIVAHHQLLAHAKVVDLYRKNY-THQGGKIGPTMITRWFLPYN-DTDRHSIAATE
 ● ●

PstrMyr 268 RYYEFECGMYFHPIS--KGDWPDIVKKRIAHRSQLEGRSSSRLPQITPEDIRLMKGAQDY
BbraMyr 246 RANQFERGWFGHPVY--KGDYPPIMKKWVDQKSKEEGLPWSKLPKFTKDEIKLLKGTADF
CnasMyr2 270 LALQFYLGWFAHPIFSKNGDYPQVMIDRIEAHSKMQGFPRSRLPTFTAEEIERIRGTSDF
CnasMyr3 270 LALQFYLGWFAHPIFSKNGNYPQVMIDRIDANSKQQGFPRSRLPKFTPEEIKRIRGTSDF
CnasMyr1 266 REMQFTLGWIAHPIFSSDGDYPAVMIDRIANHSKAEGRLVSRLPTFSNHWKRTIQGTSDF
CnasMyr4 268 EAIQFTLGFSAHPIFSTKGDYPPFVIERIYNASKEEGRLRSRLPTFSEYWIKKIKGSSDF
SalbMyr 277 RMKQFFLGWFMGPLT--NGTYPQIMIDTV----------GARLPTFSPEETNLVKGSYDF

◊
PstrMyr 326 IAINHYFTIMAANAEEAS---YDE-----INYKHDVRVNLFND-----PSWK---I--S-
BbraMyr 304 YALNHYSSRLVTFGSDPN-----------PNFNPDASYVTSVD-----EAWL---K-PN-
CnasMyr2 330 FGINSYTTVLVTRNDHNNSANFPI-----PSFNHDMGVVESVD-----ENWT---K--S-
CnasMyr3 330 FGINSYTTVLVTRNDCNNSANFPI-----PSFNHDMDVVESFD-----ENWT---K--S-
CnasMyr1 326 FGLNYYTSRYIRESLEPI---GEN-----PSVARDRGNERFTK-----PEWI---K--G-
CnasMyr4 328 FGLNYYTSRYVSKSSQPI---VDNYTTIDSILHSDLNLRLSAK-----SEGI---K--S-
SalbMyr 325 LGLNYYFTQYAQPSPNPV--NATN-----HTAMMDAGAKLTYINASGHYIGPLFESDGGD
 ● ●

▼
PstrMyr 367 VLGW-AICPFGVRELLKHLKEQYGNEPIIFAEIGVADDGT-----SLKDDIRTEYYQGYF
BbraMyr 343 ETPYIIPVPEGLRKLLIWLKNEYGNPQLLITENGYGDDG------QLDDFEKISYLKNYL
CnasMyr2 374 ASVWLHPVPSGMYNLLMWINKEYDNPPVIITENGVSDNN------GVNDMDRVEYLNSYL
CnasMyr3 374 ASFWLHPVPSGMYNLLMWINKEYDNPPVIITENGVSDNN------GVNDTDRVTYFNSYL
CnasMyr1 367 ASDWLYSVPKGLGDLLRWIKAEYNNPEVIITENGWSDDD------RLEDDSRVTYFHDHL
CnasMyr4 374 ASNRLYSVPEGLGDILKWIKAEYNNPEVIMTENGWPDDG------QLEDDGRIAYFHDHL
SalbMyr 378 GSSNIYYYPKGIYSVMDYFKNKYYNPLIYVTENGISTPGSENRKESMLDYTRIDYLCSHL

◊

PstrMyr 421 CYILEAMQIDDVNVVAIIPWSLMDNLEWAKGYIYHFGLIGIDFYNDPTLTRRLKDSTYFY
BbraMyr 397 NATLQAMYEDKCNVIGYTVWSLLDNFEWFYGYSIHFGLVKIDF-NDPQRTRTKRESYTYF
CnasMyr2 428 SSILDAMD-DGCKIQGYIAWSLMDSYEWRAGFSEQFGLYHVDF-SSPEKTRTPKASAKVY
CnasMyr3 428 ASILDAMK-DGCKIMGYIAWSLMDSFEWTAGFSEQFGLYHVDF-SSPNKTRTPKASAKMF
CnasMyr1 421 EQVLDVILNNGCNVKAFSVWSLVDNFEWSQGYTEKFGMFFVNM-SSPRKERIPKKSAYFM
CnasMyr4 428 EKVLDAIQNDDCNVKGYTAWSLIDNFEWHSGYTRKFGLFSVNM-SSPRKERIPKKSTRFM
SalbMyr 438 CFLNKVIKEKDVNVKGYLAWALGDNYEFNNGFTVRFGLSYINW-NNV-TDRDLKKSGQWY
 ●

PstrMyr 481 KNVTTTKRINCDNVK---------------------------------------------
BbraMyr 456 KNVVSTGKP---------------------------------------------------
CnasMyr2 486 AHIIRHHAVDWEY--RPELSIIKYPSVIQAQTSGHSSNSSSAAITSPIAILLAALVQYSI
CnasMyr3 486 ARIVRHHAIDWKGPDRPEPSIIKHPSIIHEQIINNSSKSSSRA-----KIVLTK--KFEV
CnasMyr1 480 EELIKKRSIPGI------------------------------------------------
CnasMyr4 487 KTLIETRKIPELH-----------------------------------------------
SalbMyr 496 QKFISP------------------------------------------------------


PstrMyr ------
BbraMyr ------
CnasMyr2 544 HLFLRK
CnasMyr3 539 HD----
CnasMyr1 ------
CnasMyr4 ------
SalbMyr ------

**Supplementary Figure 3:** Multiple sequence alignment of the myroinsases investigated in this study. Signal peptides are indicated with bold, italic letters. ◊ indicate residues involved in glucose binding. ▼ indicate catalytic sites (1^st^ – acid catalysis in Beta-glucosidase, 2^nd^ – catalytic nucleophile). ● indicate the aglycone recognition site in *Sinapis alba* myrosinase. The roles proposed are based on Burmeister et al. 1997. The threshold for similarity (grey background) and identity (black background) was set to 70%. Pstr = *Phyllotreta striolata*, Bbra = *Brevicoryne brassicae*, Cnas = *Contarinia nasturtii*, Salb = *Sinapis alba*. For further sequence information refer to Supplementary Table 3.

CnasSulf1a 1 M-------NLYQSSGTSLLVYCEII--FLVGFCMTKNHGSAHKIKGKKPNIIIIVADDMG
CnasSulf1b 1 M-------NLYQSSGTSLLVYCEII--FLVGFCMTKNHGSAHKIKGKKPNIIIIVADDMG
CnasSulf1e 1 M-------NLYQSSGTSLLVYCEII--FLVGFCMTKNHGSAHKIKGKKPNIIIIVADDMG
CnasSulf1d 1 M-------NLYQSSGTSLLVYCEII--FLVGFCMTKNHGSAHKIKGKKPNIIIIVADDMG
CnasSulf1c 1 M-------NLYQSSGTSLLVYCEII--FLVGFCMTKNHGSAHKIKGKKPNIIIIVADDMG
CnasSulf2 1 MCKRIPPGKSTLELKTFFATTILAC--II------MTKIVNCDEIEKRPHIIFILADDMG
PchrGSS1 1 M----------------MLRWCVFL--LLFGI---------GLMHTQKPNIIIIMADDMG
PchrGSS2 1 M----------------VLRWCVFF--LIFGV---------SVIHAQKPNIIIIMADDMG
PchrGSS3 1 M----------------VLRWCMIH--LLFGV---------GLMDTQKPNIIVIMADDLG
PchrGSS4 1 M----------------VLRWFMLH--LLFGV---------GLMDTQKPNIIVIMADDMG
PchrGSS5 1 M----------------ALRWFMLH--LLFGV---------GLMDTQKPNIIVIMADDMG
PxylGSS1 1 -------------------------MAILHQAVV-LLGAALCVSAATKPHVIMIMADDMG
PxylGSS2 1 -------------------------MAILHQVVV-ILGAALCVSASTKPHVIMIMADDMG
PxylGSS3 1 -------------------------MGRLVQICLVLSVALAASAQKAKPNVLFVMADDMG

▼ ▼ ▼
CnasSulf1a 52 FNDASIHGSNQIPTPNIDALGIMGVQLNRHYTAPMCTPSRSSLLTGKYESNLGMQHFVIP
CnasSulf1b 52 FNDASIHGSNQIPTPNIDALGIMGVQLNRHYTAPMCTPSRSSLLTGKYESNLGMQHFVIP
CnasSulf1e 52 FNDASIHGSNQIPTPNIDALGIMGVQLNRHYTAPMCTPSRSSLLTGKYESNLGMQHFVIP
CnasSulf1d 52 FNDASIHGSNQIPTPNIDALGIMGVQLNRHYTAPMCTPSRSSLLTGKYESNLGMQHFVIP
CnasSulf1c 52 FNDASIHGSNQIPTPNIDALGIMGVQLNRHYTAPMCTPSRSSLLTGKYESNLGMQHFVIP
CnasSulf2 53 FNDVGFHGSAQIPTPNIDALAYSGLILNNYYVTPICTPSRSALMTGKYPIHTGMQHRVLY
PchrGSS1 34 FNDVGFHGSDEIPTPNIDALAYNGVILNTHYTQSLCTPSRAAFLTGKYPIHTGMQHRVIL
PchrGSS2 34 FNDVGFHGSDEIPTPNIDALAYNGVILNTHYTQSACSPSRAAFLTGKYPIHTGMQHTVIL
PchrGSS3 34 FNDVGFHGSDEIPTPNIDALAYNGVILNTHYTQALCTPSRAAFLTGKYPIHTGMQHLVVL
PchrGSS4 34 FNDAGFHGSDEIPTPNIDALAYNGVILNRHYTQALCSPSRAAFLTGKYPIHTGMQHFVVL
PchrGSS5 34 FNDAGFHGSDEIPTPNIDALAYNGVILNKHYTQALCTPSRAAFLTGKYPIHTGMQHFVIL
PxylGSS1 35 WDDTSTHGSKSVLTPNLDVLTRSGVSLHRYYTHALCSPARTAVLTGKYAHTVGMQGMPLS
PxylGSS2 35 WDDTSTHGSKSVLTPNLDVLTRSGVSLHRYYTHAVCSPARTAVLTGKYAHTLGMQGMPLS
PxylGSS3 36 WDDWSSHGSRQVLTPNLDSLAGSGVLLHRYYTHALCSPARSAVLTGRYSHTIGMQGRPLL

▼ ▼
CnasSulf1a 112 SNAPYGLDPCEKTMANYMKDGGYRTYLVGKWHLGFFEKRYTPLYRGFDSHFGYLGPYIDY
CnasSulf1b 112 SNAPYGLDPCEKTMANYMKDGGYRTYLVGKWHLGFFEKRYTPLYRGFDSHFGYLGPYIDY
CnasSulf1e 112 SNAPYGLDPCEKTMANYMKDGGYRTYLVGKWHLGFFEKRYTPLYRGFDSHFGYLGPYIDY
CnasSulf1d 112 SNAPYGLDPCEKTMANYMKDGGYRTYLVGKWHLGFFEKRYTPLYRGFDSHFGYLGPYIDY
CnasSulf1c 112 SNAPYGLDPCEKTMANYMKDGGYRTYLVGKWHLGFFEKRYTPLYRGFDSHFGYLGPYIDY
CnasSulf2 113 AAEPRGLPLTEKILPQYLKELGYTNHCVGKWHLGHYKSEYTPLHRGFDSHIGYWTGHHDY
PchrGSS1 94 ESEPYGLPLNETLMPQLLKQNGYATHAVGKWHLGFSKKEYTPTFRGFDTHYGYWLMFHDY
PchrGSS2 94 ESEPWGLPLNETLMPQLLKQNGYVTHAVGKWHLGFFKKEYTPIFRGFDTHYGYYLMFHDY
PchrGSS3 94 EMEPWGLPLNETIMPQLLKQNGYTTHAVGKWNLGFFKKEYTPTFRGFDTHYGYWQGFHDY
PchrGSS4 94 EMEPWGLRLNETIMPQLLKQNGYTTHAVGKWNLGFFKKEYTPTFRGFDTHYGYWQGFHDY
PchrGSS5 94 EMEPWGLPLNETIMPQLLKQNGYTTHAVGKWNLGFFKKEYTPTFRGFDTHYGFWQGFHDY
PxylGSS1 95 NAEERGIPLEERLISQYLQDAGYRTQMVGKWHVGHAFFEQLPTYRGFENHFGVRGGFIDY
PxylGSS2 95 NAEERGIPLEERLISQYLQDAGYRTQIVGKWHVGHAFFEQLPTYRGFENHFGVRGGFIDY
PxylGSS3 96 HGEARGIPTTERLLPQYLKELGYRTQLVGKWHVGHAYRNQLPINRGFENHYGARTGFMDY


CnasSulf1a 172 FNHSLSIVPIPEMASGYDMRKNLSVHWDTINQYATDLFTDKAIETIQTHDKNKPMFMMLS
CnasSulf1b 172 FNHSLSIVPIPEMASGYDMRKNLSVHWDTINQYATDLFTDKAIETIQTHDKNKPMFMMLS
CnasSulf1e 172 FNHSLSIVPIPEMASGYDMRKNLSVHWDTINQYATDLFTDKAIETIQTHDKNKPMFMMLS
CnasSulf1d 172 FNHSLSIVPIPEMASGYDMRKNLSVHWDTINQYATDLFTDKAIETIQTHDKNKPMFMMLS
CnasSulf1c 172 FNHSLSIVPIPEMASGYDMRKNLSVHWDTINQYATDLFTDKAIETIQTHDKNKPMFMMLS
CnasSulf2 173 FDHTAV----EQ-QWGFDIRRGMDVAYDLHGQYTTDIITSESVRIIRSHNASHPLFLYIA
PchrGSS1 154 FTHMTKAH--LSNDTGYDFRRNLDVDWDAKGKYSTTLFTNGAVKLISKHNTNNPMFLYLA
PchrGSS2 154 YTHMTKAD--YANDTGYDFRRNLDVDWDAKGKYSTTLFTNEAVKLIREHDTNNSMFLYLA
PchrGSS3 154 YTHMNKAS--WSTEIGYDFRRNLEVDYDAKGKYSTTLFTNEAVKLIHEHDTNNPMFLYLA
PchrGSS4 154 YTHMNKAS--WSTEIGYDFRRNLDVDYDAKGKYSTTLFTNEAVKLIKEHDTTNPMFLYLA
PchrGSS5 154 YTHMNKAS--WSTEIGYDFRRNLDVDYDAKGKYSTTLFTNEAVKLIQEHDTNNPMFLYLA
PxylGSS1 155 YEYNAQEQLDGRPVTGLCLFDDLQPDWT-TEGYITDVYTEKSTTIIENHNVSEPLYLLLT
PxylGSS2 155 YEYNAQSRLDGKPVTGLCLFDDLKPDWT-TEGYITDVYTEKSTTIIENHNVSEPLYLLLT
PxylGSS3 156 YEYNSQEAWASGPVSGLSLFRDYTPDFE-AEGYITDLYNEEAKHIIRSHNTSEPLFLMVT

CnasSulf1a 232 HLAPHTANEFDPMQAPEDEINKFEYIKNEKRRVYAAMVSKLDEGVGKVVKALDQNKMLEN
CnasSulf1b 232 HLAPHTANEFDPMQAPEDEINKFEYIKNEKRRVYAAMVSKLDEGVGKVVKALDQNKMLEN
CnasSulf1e 232 HLAPHTANEFDPMQAPEDEINKFEYIKNEKRRVYAAMVSKLDEGVGKVVKALDQNKMLEN
CnasSulf1d 232 HLAPHTANEFDPMQAPEDEINKFEYIKNEKRRVYAAMVSKLDEGVGKVVKALDQNKMLEN
CnasSulf1c 232 HLAPHTANEFDPMQAPEDEINKFEYIKNEKRRVYAAMVSKLDEGVGKVVKALDQNKMLEN
CnasSulf2 228 HAAVHSGNPYNPLPAPDSTTSKLTHINDFARRKYAAMLTHLDYSVGAVVHELAQNNMLKD
PchrGSS1 212 HIAPHAGNDANPLQAPVEEIAKFAHIKDPERRNYAAMVSMLDQSVGTVIEALQEKQMLQN
PchrGSS2 212 HIAPHTANEADPLQAPDEEVAKFAHIKDPKRRVYAAMVSMLDQSVGTVIEALQEKQMLQN
PchrGSS3 212 HMAPHTGNEGEQLQAPDEEIAKFAHIKDPERRIYAAMVSMLDQSVGTVIAALREKRMLQN
PchrGSS4 212 HIAPHTGNKEEPLQAPDEEIAKFAHIKDPERRIYAAMVSMLDQSVGTVIAALREKRMLQN
PchrGSS5 212 QIAPHTANEEEQLQAPDEEIAKFAHIKDPERRIYAAMVSLLDQSVGTVIAALREKRMLQN
PxylGSS1 214 HHAPHNGNEDASLQAPPEEVRAQRHVELHPRRIFAAMVKKLDDSIGEIVATLEKKGMLEN
PxylGSS2 214 HHAPHNXNEDASLQAPPEEVRAQRHVELHPRRIFAAMVKKLDDSIGEIVATLEKKGMLEN
PxylGSS3 215 HHAPHNGNEDASLQAPPEEVRAQRHVELHPRRIFAAMVKKLDDSVGDMVATLKEQGMLEN


CnasSulf1a 292 SIILFFSDNGSPIVGEHANAGSNFPFKGQKDSPWEGATRNLAAIWSL-HLKR----RQRV
CnasSulf1b 292 SIILFFSDNGSPIVGEHANAGSNFPFKGQKDSPWEGATRNLAAIWSL-HLKR----RQRV
CnasSulf1e 292 SIILFFSDNGSPIVGEHANAGSNFPFKGQKDSPWEGATRNLAAIWSL-HLKR----RQRV
CnasSulf1d 292 SIILFFSDNGSPIVGEHANAGSNFPFKGQKDSPWEGATRNLAAIWSL-HLKR----RQRV
CnasSulf1c 292 SIILFFSDNGSPIVGEHANAGSNFPFKGQKDSPWEGATRNLAAIWSL-HLKR----RQRV
CnasSulf2 288 SIIVFSTDNGGPAEGFNLNAASNWPLRGVKNTLWEGGVRGAALLFSP-MLTK----KNRV
PchrGSS1 272 SVILFLSDNGATK------YGSNYPFKGLKESSWEGANRNLAAIWSP-LIQK----SQRV
PchrGSS2 272 SVILFLSDNGPTK------YGSSYPLKGIKYSSWEGGNRNLAAIWSP-LIQK----SQRV
PchrGSS3 272 SVILFFSDNGAAQ------FGSSYPLRGKKRSAWEGGNRNLAAIWSP-LIQK----PQRV
PchrGSS4 272 SVILFFSDNGAAD------FGSNYPFRGKKQTAWEGGNRNLAAIWSP-LIQK----PQRV
PchrGSS5 272 SVILFFSDNGAAE------FGSNYPFRGKKNTAWEGGNRNLAAIWSP-LIQK----PQRV
PxylGSS1 274 TIITFSTDNGAPTVGLGANSGSNYPLRGVKKSPWEGGIRGNAMIWAGPEVAPGNAWRGKV
PxylGSS2 274 TIITFSTDNGAPTVGLGANSGSNYPLRGVKKSPWEGGIRGNAMIWAGPEVAPGNSWRGNV
PxylGSS3 275 TIIVFVADNGAPTVGNGANSGSNYPLRGVKGSPWEGGIRVDALVWAGPEVAEGNDWRGNI


CnasSulf1a 347 SNEMFHISDWLPTFAKIAGFNIEG---PIDGKNIWNSLSYDLPSPRRDVLLHHDPEVP--
CnasSulf1b 347 SNEMFHISDWLPTFAKIAGFNIEG---PIDGKNIWNSLSYDLPSPRRDVLLHHDPEVP--
CnasSulf1e 347 SNEMFHISDWLPTFAKIAGFNIEG---PIDGKNIWNSLSYDLPSPRRDVLLHHDPEVP--
CnasSulf1d 347 SNEMFHISDWLPTFAKIAGFNIEG---PIDGKNIWNSLSYDLPSPRRDVLLHHDPEVP--
CnasSulf1c 347 SNEMFHISDWLPTFAKIAGFNIEG---PIDGKNIWNSLSYDLPSPRRDVLLHHDPEVP--
CnasSulf2 343 SKQKLHIVDWLPTLYYAAGGNVEQLNVTLDGKNLWPALSEDTKSDRTEVLHNIDDIFG--
PchrGSS1 321 SNHLMHISDWLPTFYSIAGLNKTQIP-NIDGLDMWESISEDKESPRTEMLYNIDDTAR--
PchrGSS2 321 SNRLMHISDWLPTFYSIAGLNKTQIP-NIDGQDMWESISEDKESPRTEMLYNIDDVTTGW
PchrGSS3 321 SNQLIHIIDWLPTFYSIAGLNKTEIS-NTDGIDMWESISEDKESPRIEMVYNIDTIRK--
PchrGSS4 321 SNRLIHIIDWLPTFYSIAGLNKTEIL-NTDGLDMWESISEDKESPRTEMVYNIDTIRK--
PchrGSS5 321 SNHLIHVTDWLPTFYSIAGLNKTQIP-NIDGLDMWESISKDKESPRTEMVYNIDTIRK--
PxylGSS1 334 YDGNMHAADWVPTLLEAIGEKI--PA-GLDGIPMWSHIIENKPSPRTEI-FEIDDYFN--
PxylGSS2 334 YDGNMHAADWVPTLLEAVGEKI--PA-GLDGIPMWSHTIENXPSPRTEI-FEIDDYFN--
PxylGSS3 335 FQGKMHASDWLPTLLEAIGEKP--PT-GIDGIPQWKHILENQPT-RQEI-FEIDDFTG--


CnasSulf1a 402 --YMAYISENLKLVSGSTYDGM----YDKWLSEPIDQSEENSTFGEKYSEAILSSNVGQV
CnasSulf1b 402 --YMAYISENLKLVSGSTYDGM----YDKWLSEPIDQSEENSTFGEKYSEAILSSNVGQV
CnasSulf1e 402 --YMAYISENLKLVSGSTYDGM----YDKWLSEPIDQSEENSTFGEKYSEAILSSNVGQV
CnasSulf1d 402 --YMAYISENLKLVSGSTYDGM----YDKWLSEPIDQSEENSTFGEKYSEAILSSNVGQV
CnasSulf1c 402 --YMAYISENLKLVSGSTYDGM----YDKWLSEPIDQSEENSTFGEKYSEAILSSNVGQV
CnasSulf2 401 --SASLTVGEWKIHKGTNYNGA----WDSWYGPA-GIRSAT----AYNVNGVIQSPAGQV
PchrGSS1 378 --WGAIRQGDWKYIYGSTGTEK-----DLWYGND-GKKPEY----SYDVNQILSSKSATA
PchrGSS2 380 EAWGAIRQGDWKYIYGSTGNEK-----DSWYGND-GKRPEY----SYDINQILTSKTAAA
PchrGSS3 378 --WGAIRQGDWKYIYGSTGKEK-----NSWFGND-GKKKEY----SYDIDQILTSKTATA
PchrGSS4 378 --WGAIRQGDWKYIYGSTGKEK-----NSWFGND-GKKKEY----SYDVDQILTSKTAAA
PchrGSS5 378 --WGAIRQGDWKYIYGSTRTKK-----NSWFGND-GKKKEY----SYDVDQILTSKTATA
PxylGSS1 388 --HSSVTLGRHKLVKGTIDESLSKHY-----GEDLRGIIGTP---PDYKQKLRDSKAWES
PxylGSS2 388 --HSSVTLGRHKLIKGTIEESLSKHY-----GEDLRGIIGTP---PDYKQKLXDSKAWES
PxylGSS3 388 --YSSITLGRHKLIVGTVNAAYNLHR-----GGDLRGIIGQP---PNYQQAVLNSKAYAA

CnasSulf1a 456 LQKYSTSN--------RNKFYNYVETDSHTISADEINEIRFKAKVTCNGNIPPNNNNSVA
CnasSulf1b 456 LQKYSTSN--------RNKFYNYVETDSHTISADEINEIRFKAKVTCNGNIPPNNNNSVA
CnasSulf1e 456 LQKYSTSN--------RNKFYNYVETDSHTISADEINEIRFKAKVTCNGNIPPNNNNSVA
CnasSulf1d 456 LQKYSTSN--------RNKFYNYVETDSHTISADEINEIRFKAKVTCNGNIPPNNNNSVA
CnasSulf1c 456 LQKYSTSN--------RNKFYNYVETDSHTISADEINEIRFKAKVTCNGNIPPNNNNSVA
CnasSulf2 450 LNRINL-----------------------LPSAVHMRKIRSDATVDCTK-----NQTSES
PchrGSS1 426 FAGLITYQQIRDKNSNTK-----RRRELKIIDKNDIEMLRKQATVHCGPFT-FEYQLEEN
PchrGSS2 430 FAGLITYQQI--KMESTK-----EGKELKIIDKNDIEMLRMQATVRCGPFN-FEDQPEEN
PchrGSS3 426 FAGLITDQQVKDKNSNRR-----KRRELKIIDQNDIETLRTQATVTCGPFN-FEDQPEEN
PchrGSS4 426 FAGLITDQQIKDKHSNRR-----KRRELKIIDKNDIETLRTQAIVTCGPFN-FEDQLEEN
PchrGSS5 426 FAGLIADQQINDKNSNRK-----KRRELKIIDKNDIETLRTQATVTCGPFN-FEDQQEEN
PxylGSS1 438 LETIGI-----------------------PLDAD-VMADRDEAIVTCGNVVP--------
PxylGSS2 438 LETIGI-----------------------PLDAD-VMADRDEAIVTCGDVVP--------
PxylGSS3 438 ITAAGV-----------------------PIDVEHLEENKEKATVTCGEGVA--------


CnasSulf1a 508 -ACNP-IISPCLFNISDDPCETTNIAAQFPDIVKKLESKLDYYCEIAKPIRN---KPGDP
CnasSulf1b 508 -ACNP-IISPCLFNISDDPCETTNIAAQFPDIVKKLESKLDYYCEIAKPIRN---KPGDP
CnasSulf1e 508 -ACNP-IISPCLFNISDDPCETTNIAAQFPDIVKKLESKLDYYCEIAKPIRN---KPGDP
CnasSulf1d 508 -ACNP-IISPCLFNISDDPCETTNIAAQFPDIVKKLESKLDYYCEIAKPIRN---KPGDP
CnasSulf1c 508 -ACNP-IISPCLFNISDDPCETTNIAAQFPDIVKKLESKLDYYCEIAKPIRN---KPGDP
CnasSulf2 482 -ICRP-LEKPCLFNVDEDPCEQNNLAEQNPQILNDLLERLQYYNQTAIPPGN---LPLDP
PchrGSS1 480 -ECNL-NQSPCLFNIREDPCERVNLASTAPNIVKNLEQLLLEYRKNIVPALN---RPRDP
PchrGSS2 482 -KCNL--KSPCLFNIKEDPCERVNLASARPDILKNLETLLLDYRKNMVPALN---KPRDP
PchrGSS3 480 -KCNL--QLPCLFNIKEDPCERVNLAKSRPNIVRNLEKLLVDYRKNIVPALN---ADRDP
PchrGSS4 480 -KCNL--QLPCLFNIKEDPCERVNLAKSRPNIVRKLEKLLMDYRKNIVPALN---EDRDP
PchrGSS5 480 -KCNL--QLPCLFNIKQDPCERVNLAKSRPNIVRKLEKLLLDYSKNILPALN---KDRDP
PxylGSS1 466 KPCSPSAESWCLYDIIEDPCELRDLSEELPQLAQILLYRLEQEEAKIIPREGQ--YVADP
PxylGSS2 466 KPCSPTAESWCLYDIIADPCELRDLSEELPQLAQILLYRLEQEEAKIIPRDGQ--FVADP
PxylGSS3 467 KECIPTADKWCLYDIIDDPCEYRDLSDSLPGLAEVLRFRLAQEEERVVPRTDKNDPIGSE


CnasSulf1a 563 RSNPANFGGIWTWWYDELNITTQSTA-------------WRMGEHSLQTHIPFHICVAIV
CnasSulf1b 563 RSNPANFGGIWTWWYDELNITTQT---------------WRMGEHSLQTHIPFHICVAIV
CnasSulf1e 563 RSNPANFGGIWTWWYDELNITTQSTG--------------------------------KV
CnasSulf1d 563 RSNPANFGGIWTWWYDELNITTQN---------------CCNDHHKKD----------GV
CnasSulf1c 563 RSNPANFGGIWTWWYDELNITTQSTD-------------CCNDHHKKD----------GV
CnasSulf2 537 NADPRFFDNTWTNFGDFNKKMMYS------------------------------------
PchrGSS1 535 RSNPINWNNTWTNWEDYDQDFRSRVINNRIVN-----SREINSNKPISS---INIS-III
PchrGSS2 536 RSNPIYWNNTWTYWQDYDQVVGSRVINNTIINSTVINSTVINSNKPIST---INII-III
PchrGSS3 534 RANPINWNNTWSSWLDSDYIVRSKV---------------PNSFRTIST---MNII-IIV
PchrGSS4 534 RANPINWNNTWSSWIDSDYVARSKV---------------PNSFKTTST---MNII-IIV
PchrGSS5 534 RVNPINWNNTWSSWLDSDYVAGGKL---------------PNSFKTTSA---MNII-IIA
PxylGSS1 524 KSAPKYFNYTWDAYLSVEPYSDSE------------------------------------
PxylGSS2 524 RAAPKYFNYTWDSYLSVEPYSDSE------------------------------------
PxylGSS3 527 RAMSKNFNYTWETFMDLEPYTA--------------------------------------


CnasSulf1a 610 AMIITRKMAWL---------------------------------
CnasSulf1b 608 AMIITRKMAWL---------------------------------
CnasSulf1e 591 KILLQ---------------------------------------
CnasSulf1d 598 ALKISSIY------------------------------------
CnasSulf1c 600 ALKISSIY------------------------------------
CnasSulf2 --------------------------------------------
PchrGSS1 586 VSSILILFMVFGL-SKLADIEARRRE----ERNADALLRNNNS-
PchrGSS2 592 LSSIMILFMIFGL-SKLADTEENRRERKEQKRKTKALLSNNNYF
PchrGSS3 575 VSSIIILFIVFRLSSKLADTKAKKRE---QKRKAETLLSNNSV-
PchrGSS4 575 VSSITILFIVFRLSSKLSDTKAKKRE---QKRKAETLLSNNSV-
PchrGSS5 575 VSSITILFIVFRLSSKLSDTKAKKRE---QKQKDKTFLSNNSV-
PxylGSS1 --------------------------------------------
PxylGSS2 --------------------------------------------
PxylGSS3 --------------------------------------------

**Supplementary Figure 4:** Multiple sequence alignment of the arylsulfatases (Sulf) and glucosinolate sulfatases (GSS) investigated in this study. The two highly conserved motifs that are recognized as signature sulfatase features are indicated with a bar above the alignment and the catalytic residues are indicated with ▼ (Hanson et al. 2004). Signal peptides are in bold, italic letters, and transmembrane domains are double underlined. The threshold for similarity (grey background) and identity (black background) was set to 70%. Cnas = *Contarinia nasturtii*, Pchr = *Psylloides chryosocephala*, Pxyl = *Plutella xylostella*. To simplify the alignment *CnasSulf1f*-*i* and *CnasSulfk-l* were removed as their sequences are identical to *CnasSulf1a* and *CnasSulf1c*, respectively. For further sequence information refer to Supplementary Table 4.

* ***

CnasGST11a 1 -MDFYYIPGSAPCRAVQMTAKAVGVELNLKLTNLMAGEHLKPEFIKINPQHTVPTLVDTK
CnasGST11b 1 -MDFYYIPGSAPCRAVQMTAKAVGVELNLKLTNLMAGEHLKPEFIKINPQHTVPTLVDTK
SnigGSTD1a 1 MADLYYLPGSSPCRSVIMVAKAIGLELNKKLLDLSTGEHLKPEFVKINPQHTIPTLVDN-
SnigGSTD1b 1 MADLYYLPASASCRSVIMVAKAIGLELNKKLLDLNTGEHLKPEFVKINPQHTIPTLVDN-
SflaGSTD1 1 MADFYYLPGSAPCRSVIMVAKAIGLELNKKILDLHAGEHLKPEFIKLNPQHTIPTLVDN-

 **
CnasGST11a 60 ENLNLWESRAIMVYLVEKYGKTASLYPADPKARALVNQRLYFDLGTLYQRLADYYYPQIF
CnasGST11b 60 ENLNLWESRAIMVYLVEKYGKTASLYPADPKARALVNQRLYFDLGTLYQRLADYYYPQIF
SnigGSTD1a 60 -GFALWESRAILVYLVEKYGKTDSLYPKCPKKRAVINQRLYFDMGTLYQSFANYYYPQVF
SnigGSTD1b 60 -GFALWESRAILVYLVEKYGKTDSLYPKCPKKRAVINQRLYFDMGTMYQSFGDYYYPQLF
SflaGSTD1 60 -GFALWESRAILVYLVEKYGKTDSLYPKCPKKRAVINQRLYFDMGTLYQSLSNYYYPQLF

 **●** ● **◊**
CnasGST11a 120 AKAPANPENLKKLEDAVGFLNTFLEGQTYAAGDNLTVADISLVATVSTLDVAGFDLSKYP
CnasGST11b 120 AKAPANPENLKKLEDAVGFLNTFLEGQTYAAGDNLTVADISLVATVSTLDVAGFDLSKYP
SnigGSTD1a 119 AKAPADPELYKKMEAAVEFLNTFLEGQTYAAGDSLTIADIALLATMSSFEVAGYDFSKYE
SnigGSTD1b 119 GKAPADPELYKKIGVAFELLNTFLEGQTYAAGDSLTIADIALLATVSTFEVAGYDFSKYE
SflaGSTD1 119 AKAPADPELYKKMGDAFGFLNTFLDGQTYAAGDSLTIADIALLASVSTFEVAGYDFSKYE

 α
CnasGST11a 180 NVVAWYEKCKATTPGYDINTAGLAEFKKFFN
CnasGST11b 180 NVVAWYEKCKATTPGYDINTAGLAEFKKFFN
SnigGSTD1a 179 NVNKWYANAKKVTPGWDENWAGCQEFKKYFE
SnigGSTD1b 179 NVNKWYANAKKVTPGWDENWAGCQEFKKYFG
SflaGSTD1 179 NVSKWYANAKKVTPGWEENWAGCLEFKKFFD

**Supplementary Figure 5:** Multiple sequence alignment of the delta class glutathione S-transferase investigated in this study that cluster with those identified in *Scaptomyza flava* and *S. nigrita*. The highly conserved insect type I glutathione s-transferase G-site is indicated with an * (Ranson et al. 1997). Sites marked with an ●, ◊, α indicate amino acids of interest in the aromatic zipper motif, H-site and α8-helix, respectively, identified by Gloss et al. (2014) to be important for isothiocyanate detoxification. The threshold for similarity (grey background) and identity (black background) was set to 70%. Cnas = *Contarinia nasturtii*, Sfla = *Scaptomyza flava*, Snig = *Scaptomyza nigrita*. For further sequence information refer to Supplementary Table 5.

**Additional References**

Andrews, S. FastQC: a quality control tool for high throughput sequence data. Available online: <http://www.bioinformatics.babraham.ac.uk/projects/fastqc>

Bolger AM, Lohse M, Usadel B. 2014. Trimmomatic: a flexible trimmer for Illumina sequence data. Bioinformatics 30(15):2114-2120.

Burmeister WP, et al. 1997. The crystal structures of *Sinapis alba* myrosinase and a covalent glycosyl–enzyme intermediate provide insights into the substrate recognition and active-site machinery of an S-glycosidase. Structure 5(5):663-676.

Challis R, Richards E, Rajan J, Cochrane G, Blaxter M. 2020. BlobToolKit–Interactive quality assessment of genome assemblies. G3 (Bethesda) 10(4):1361-1374.

Gloss AD, et al. 2019. Evolution of herbivory remodels a *Drosophila* genome. *bioRxiv*: 767160.

Guindon S, et al. 2010. New algorithms and methods to estimate maximum likelihood phylogenies: assessing the performance of PhyML 3.0. Syst Biol. 59(3):307–321.

Hanson SR, Best MD, Wong CH. 2004. Sulfatases: structure, mechanism, biological activity, inhibition, and synthetic utility. Angew Chem Int. 43:5736-5763.

Kalyaanamoorthy S, Minh BQ, Wong, TKF, von Haeseler A, Jermiin, LS. 2017. ModelFinder: fast model selection for accurate phylogenetic estimates. Nat Methods. 14(6):587-589.

Liu J, Mori BA, Olfert O, Hallett RH. 2019. Determining temperature-dependent development and mortality parameters of the swede midge (Diptera: Cecidomyiidae). J Econ Entomol. 112(4):1665-1675.

Rambaut A, Drummond AJ. 2010. FigTree v1.4.4. <http://tree.bio.ed.ac.uk/software/figtree/> (Accessed 3 October 2020)

Ranson H, et al. 1997. Cloning and localization of a glutathione S-transferase class I gene from *Anopheles gambiae*. J Biol Chem. 272(9):5464-5468.

Simão FA, Waterhouse RM, Ioannidis P, Kriventseva EV, Zdobnov EM. 2015. BUSCO: assessing genome assembly and annotation completeness with single-copy orthologs. Bioinformatics 31(19):3210-3212.

Trifinopoulos J, Nguyen LT, von Haeseler A, Minh BQ. 2016. W-IQ-TREE: a fast online phylogenetic tool for maximum likelihood analysis. Nucleic Acids Res*.* 44(W1):W232-W235.

Waterhouse RM, et al. 2018. BUSCO applications from quality assessments to gene prediction and phylogenomics. Mol Biol Evol. 35(3):543-548.
